# High-Resolution Genomic Surveillance of Carbapenem-Resistant *Acinetobacter baumannii*: IC-2 Clonal Diversity, Resistance Determinants, and Virulence Signatures

**DOI:** 10.3390/antibiotics15050464

**Published:** 2026-05-04

**Authors:** Arianna Basile, Valentina Antonelli, Claudia Rotondo, Michele Properzi, Francesco Messina, Silvia D’Arezzo, Valentina Dimartino, Ivano Petriccione, Laura Loiacono, Maria Grazia Bocci, Giulia Capecchi, Alessia Arcangeli, Alessandra Marani, Filippo Pasquale Riggio, Massimiliano Lucidi, Francesco Imperi, Paolo Visca, Carla Fontana

**Affiliations:** 1 Department of Science, Roma Tre University, 00146 Rome, Italy; arianna.basile@uniroma3.it (A.B.); filippopasquale.riggio@uniroma3.it (F.P.R.); massimiliano.lucidi@uniroma3.it (M.L.); francesco.imperi@uniroma3.it (F.I.); paolo.visca@uniroma3.it (P.V.); 2 Microbiology and Biobank Unit, National Institute for Infectious Diseases “Lazzaro Spallanzani”, IRCCS, 00149 Rome, Italy; valentina.antonelli@inmi.it (V.A.); michele.properzi@inmi.it (M.P.); mss.francesco1984@gmail.com (F.M.); silvia.darezzo@inmi.it (S.D.); valentina.dimartino@inmi.it (V.D.); ivano.petriccione@inmi.it (I.P.); carla.fontana@inmi.it (C.F.); 3 Clinical and Research Infectious Disease Department, National Institute for Infectious Diseases “Lazzaro Spallanzani”, IRCCS, 00149 Rome, Italy; laura.loiacono@inmi.it; 4 Intensive Care Unit, National Institute for Infectious Diseases “Lazzaro Spallanzani”, IRCCS, 00149 Rome, Italy; mariagrazia.bocci@inmi.it; 5 Clinical Risk Management, National Institute for Infectious Diseases “Lazzaro Spallanzani”, IRCCS, 00149 Rome, Italy; alessia.arcangeli@inmi.it; 6 Health Direction, National Institute for Infectious Diseases “Lazzaro Spallanzani”, IRCCS, 00149 Rome, Italy; alessandra.marani@inmi.it; 7 National Biodiversity Future Center, 90133 Palermo, Italy; 8 Santa Lucia Foundation IRCCS, 00179 Rome, Italy

**Keywords:** *Acinetobacter baumannii*, antibiotic-resistance, carbapenem resistance, CRAB, epidemiology, phylogeny, surveillance, virulence factors, whole-genome sequencing

## Abstract

**Background/Objectives:** *Acinetobacter baumannii* is a critical opportunistic pathogen causing severe healthcare-associated infections, particularly in intensive care units. The global dissemination of carbapenem-resistant *A. baumannii* (CRAB) and its environmental persistence necessitate continuous genomic surveillance to monitor high-risk clones. **Methods:** We conducted whole-genome sequencing (WGS), core genome multi-locus sequence typing (cgMLST), and phylogenomic analyses on 26 CRAB isolates collected at the National Institute for Infectious Diseases (INMI) “Lazzaro Spallanzani” IRCCS (September 2023–September 2024). Antimicrobial resistance determinants, virulence-related genes, and capsular (KL) and lipooligosaccharide outer core (OCL) loci were characterized by interrogation of comprehensive bioinformatic pipelines. **Results:** All CRAB isolates displayed an extensively drug-resistant (XDR) phenotype, with a shared resistance pattern to carbapenems, aminoglycosides, fluoroquinolones, fosfomycin, and sulfonamides, while being susceptible only to colistin and cefiderocol. The carbapenemase gene *bla*_OXA-23_ was detected in all CRAB isolates, together with clone-specific *bla*_OXA-51-like_ variants. For all isolates, the resistome profile fully matched the observed resistance phenotype. All isolates belonged to the International Clonal Lineage II (ICL II), Pasteur Sequence Type (ST) 2, and Oxford ST369, ST208, and ST455. Integration of cgMLST data with phylogenomic analyses and genome-based classification of KL and OCL loci revealed five distinct clusters, each one including nearly identical isolates, indicating both intra-hospital dissemination and possible inter-hospital transmission. Virulome profiling revealed heterogeneous repertoires of virulence-associated genes, resulting in cluster-specific patterns, while patristic analysis identified phylogenetic clusters linking the study isolates to other Italian and other European lineages. **Conclusions:** This study underscores the complex genomic landscape of CRAB in our setting, driven by the circulation of different ICL II clonal types, and reinforces the urgency of integrated genomic surveillance and robust antimicrobial stewardship to mitigate the spread of high-risk XDR *A. baumannii* clones.

## 1. Introduction

*Acinetobacter baumannii* is a Gram-negative opportunistic pathogen responsible for healthcare-associated infections, particularly among critically ill patients in intensive care units (ICUs) [1]. Its clinical significance is mainly due to its ability to cause various infections, including severe ventilator-associated pneumonia, bloodstream infections, urinary tract infections, wound infections, and post-neurosurgical meningitis [2,3]. *A. baumannii* often colonises the respiratory and gastrointestinal tracts and the skin of patients who become reservoirs for transmission to other patients, facilitating silent dissemination within hospitals [4]. *A. baumannii* is particularly persistent in healthcare settings due to its tolerance to desiccation, powerful surface adhesion properties, ability to form biofilms, and effective person-to-person or indirect transmission via contaminated medical devices. This results in endemic circulation and recurrent outbreaks of infection, particularly in ICUs [2,5,6]. The clinical relevance of *A. baumannii* is exacerbated by its multidrug-resistant (MDR) or extensively drug-resistant (XDR) phenotypes, which include resistance to several antimicrobials, primarily broad-spectrum β-lactams, aminoglycosides, and fluoroquinolones [7]. Despite the substantial clinical impact of carbapenem-resistant *A. baumannii* (CRAB) infections, no therapeutic regimen has consistently produced superior outcomes, and combination therapy is often used empirically in the absence of evidence-based standards [8]. Consequently, the World Health Organization (WHO) has classified CRAB as a critical priority pathogen [9]. Over the past two decades, CRABs have become more prevalent in Europe [10]. In fact, surveillance data from the European Centre for Disease Prevention and Control (ECDC) indicate a progressive rise in carbapenem resistance since the early 2000s, resulting in endemic transmission of CRAB in several countries [11]. Italy has consistently reported the highest prevalence in Europe [12,13], and epidemiological studies have documented the widespread dissemination of CRABs, particularly within ICUs, where repeated outbreaks and long-term persistence have been documented [14]. The combination of high antimicrobial pressure, inter-hospital spread, and the remarkable adaptability of certain *A. baumannii* clones has contributed to the continued increase in CRAB incidence observed throughout Europe [15]. While multiple *A. baumannii* lineages circulate globally, only a few highly successful epidemic clones, such as International Clonal Lineage II (ICL II), are responsible for most outbreaks worldwide [15,16]. ICL II has become the predominant high-risk lineage due to its extensive antimicrobial resistance and capacity to acquire various β-lactamase genes, driven by its strong genomic plasticity [17,18,19].

In this context, genomic surveillance is an essential tool for elucidating the evolutionary dynamics and transmission pathways of CRAB in healthcare facilities, as well as tracing its global epidemiology. The integration of whole-genome sequencing (WGS) with core genome multi-locus sequence typing (cgMLST), along with resistome and virulome profiling, enables high-resolution tracking of circulating clones, the identification of emerging lineages, and the characterization of molecular determinants associated with antimicrobial resistance (AMR) and virulence [20,21]. Such data are relevant for detecting dissemination events between wards or hospitals, informing infection prevention strategies, and supporting antimicrobial stewardship interventions.

Alongside the growing insights into the molecular epidemiology of CRAB, large-scale prospective clinical studies have emphasised the high mortality rates associated with CRAB bloodstream infections, highlighting the paramount importance of prompt and appropriate antimicrobial therapy. Notably, a nationwide Italian multicentre cohort study demonstrated that regimens containing cefiderocol are associated with improved early survival and clinical success in cases of CRAB bacteraemia. However, prior colonisation and severity of illness still play a significant role in determining outcomes [22].

In this framework, integrating high-resolution genomic data with contemporary clinical evidence is crucial for interpreting the underlying biology of therapeutic responses more effectively and supporting informed antimicrobial stewardship strategies. In line with this, we aimed to characterise the genomes of 26 CRAB isolates collected at the National Institute for Infectious Diseases (INMI) ‘Lazzaro Spallanzani’ IRCCS between 2023 and 2024. By integrating MLST, phylogenomic analyses, and resistance and virulence gene profiling, we were able to trace the local circulation of high-risk clones, establish their phylogenetic correlations with prevalent clones in Europe and North Africa, and gain insight into the molecular features that could influence their virulence and persistence in the hospital setting.

## 2. Results

### 2.1. Collection, Antimicrobial Susceptibility Testing, and Antimicrobial Resistance Genes of the A. baumannii Isolates

Between September 2023 and September 2024, 152 *A. baumannii* isolates (57 in 2023 and 95 in 2024) were identified by the Microbiology Unit of the INMI “Lazzaro Spallanzani” (Rome, IT). The vast majority of the isolates were MDR (93.0% in 2023 and 95.8% in 2024). Most isolates were recovered from the ICU, increasing from 54.0% in 2023 to 69.0% in 2024. Among *A. baumannii*-positive specimens, deep respiratory samples were the most frequent (~40%), followed by bloodstream infections (~11%), and surveillance swabs (~9%).

**Figure 1:**
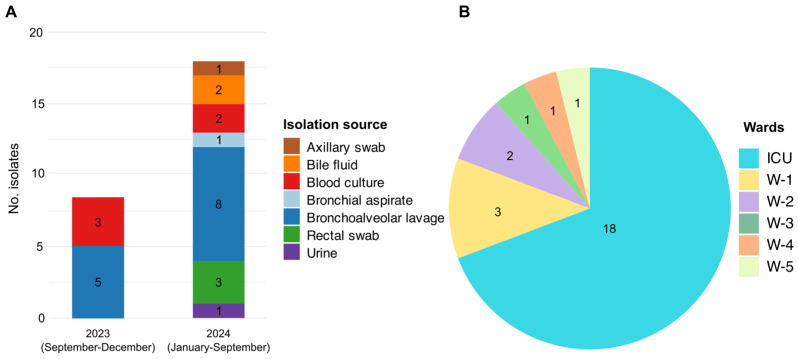
Characteristics of the *A. baumannii* clinical isolates. (**A**) Temporal distribution and isolation of the source of isolates collected over the study period. (**B**) Distribution of isolates by hospital wards. ICU: Intensive Care Unit; W-1: Post-surgical ICU; W-2: Hepatology Unit; W-3: Respiratory Diseases Unit; W-4: Immune Systemic Infectious Disease Unit; W-5: High-Intensity Care Infectious Unit.

A total of 26 CRAB isolates were selected from patients, cared for in various wards of the INMI “Lazzaro Spallanzani” IRCCS from September 2023 to September 2024. Eight isolates were collected in 2023 and 18 in 2024 (Figure 1A), all representing the first isolate obtained from individual patients. Only CRAB isolates with complete microbiological and clinical data were selected for the study, and only the first isolate from each patient was considered to avoid intra-patient duplication. Patient’s age ranged from 38 to 80 years (median age: 64). All but one patient (the one yielding Acibau-22) had previously been hospitalised in external healthcare facilities before transfer to INMI “Lazzaro Spallanzani” IRCCS wards or the ICU, and all of them were *A. baumanni*-positive at admission. All patients exhibited a severe clinical status at the time of sampling.

CRAB isolates were most frequently recovered from bronchoalveolar lavage (BAL) samples (*n* = 13; 5.0%), followed by blood cultures (*n* = 5; 19.2%), rectal swabs (*n* = 3; 11.5%), and bile fluid (*n* = 2; 7.7%). Less common sources included bronchial aspirates (BAS), urine, and axillary swabs, each represented by a single isolate (*n* = 1; 3.8%) (Figure 1A).

Ward distribution revealed a marked predominance in the ICU, which accounted for most cases (*n* = 18; 69.2%), followed by the post-surgical ICU (W-1) (*n* = 3; 11.7%), and other medical wards (W-2, W-3, W-4, and W-5) (Figure 1B).

The antimicrobial susceptibility profile, interpreted according to EUCAST 2024 (v.14.0) [23], revealed a consistent pattern of extensively drug-resistant (XDR) phenotype (Table 1). All isolates were resistant to carbapenems, including imipenem, meropenem, and ertapenem, as well as to gentamicin, amikacin, ciprofloxacin, fosfomycin, and trimethoprim–sulfamethoxazole. Susceptibility was only observed for colistin and cefiderocol, with MIC values ranging from 0.25 to 0.5 mg/L for colistin and from 0.25 to 2 mg/L for cefiderocol. Tigecycline MICs ranged from 1 to 4 mg/L, though EUCAST 2024 (v.14.0) does not provide interpretive categories for this agent. Overall, these results confirm an XDR phenotype, characterized by uniform resistance across multiple antimicrobial classes. Notably, the uniform susceptibility to cefiderocol observed across all isolates is consistent with its preserved in vitro activity against ICL II CRAB lineages circulating in Italian healthcare settings.

**Figure 2:**
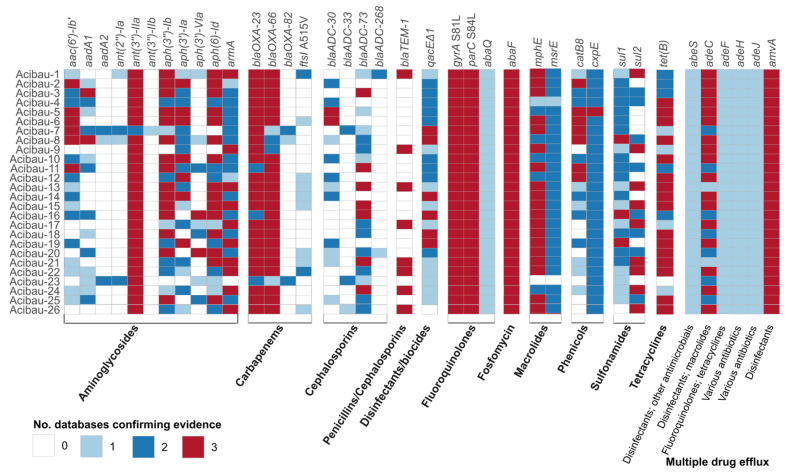
Antimicrobial resistance determinants inferred from WGS analysis of the *A. baumannii* isolates. Resistance genes were identified using three independent databases (CARD, ResFinder, AMRFinder), and the number of databases supporting the presence of each gene is reported by color code. Antibiotic classes and documented antibiotic specificity were defined based on metadata provided by the three databases and further refined through manual curation.

The 26 CRAB genomes were entirely sequenced and initially analysed to characterize the genetic basis of AMR. Interrogation of the AMRFinder [24], CARD [25], and ResFinder [26] databases for the presence of AMR genes in *A. baumannii* genomes showed that the 26 isolates harboured several antibiotic resistance genes, consistent with their XDR phenotype. All isolates carried the carbapenemase gene *bla*_OXA-23_, and either *bla*_OXA-66_ or *bla*_OXA-82_ genes, both of which are allelic variants of the intrinsic *bla*_OXA-51_ gene. Careful genome inspection indicated that all but two CRAB isolates carried *bla*_OXA-66_, while Acibau-7 and Acibau-23 carried *bla*_OXA-82_. IS*Aba1* was not detected upstream of any of the *bla*_OXA-51-like_ genes. Among the less frequently detected β-lactamase genes were *bla*_ADC-33_ (3/26 isolates; 11.6%), *bla*_ADC-30_ (13/26 isolates; 50.0%), *bla*_TEM-1D_ (8/26 isolates; 30.7%), and *bla*_ADC-268_, which was detected in only two isolates (Figure 2). Comparison of AMR gene profiles with MIC data indicates that *bla*_OXA-23_ and *bla*_OXA-66_ provide the genetic groundwork for carbapenem resistance, in association with additional β-lactamase genes and/or the A515V mutation in *ftsI*, which encodes the Penicillin-Binding Protein 3, a β-lactam target (Figure 2, Table 1).

Several isolates carried a broad array of genes conferring resistance to aminoglycosides, fluoroquinolones, fosfomycin, tetracyclines, and sulfonamides. The aminoglycoside nucleotidyltransferase gene *ant(3′′)-IIa* was detected in all isolates (Figure 2), while the 16S rRNA methyltransferase gene *armA* was present in all isolates except Acibau-23 (96.2%). Interestingly, all isolates harboured the S81L and S84L mutations in *gyrA* and *parC*, respectively, which are known to confer fluoroquinolone resistance [27], and, accordingly, all isolates were resistant to ciprofloxacin (Figure 2, Table 1). Also, the fosfomycin efflux transporter, *abaF*, was invariably detected in all isolates, mirroring their fosfomycin resistance, as expected according to EUCAST 2024 (v.14.0) guidelines (Figure 2, Table 1) [23].

Although macrolides, phenicols, tetracycline, and sulfonamides are not recommended for treating *A. baumannii* infections, macrolide resistance genes *msr(E)* and *mph(E)* were detected in all isolates but Acibau-23, while the chloramphenicol resistance gene *cpxE* was detected in all isolates. The tetracycline efflux gene *tet(B)* was present in all isolates but Acibau-23, all showing low tigecycline MICs due to the poor activity of *tet(B)* to glycylglycine’s efflux [28,29]. While *dfr* genes were not detected in any of the isolates, the sulfonamide resistance genes *sul1* and *sul2* were widely distributed, being detected in 25 (96.2%) and 17 (65.3%) of the isolates, respectively. Overall, all isolates carried at least one of these two genes in combination with multiple efflux systems (*ade* genes), consistent with their trimethoprim–sulfamethoxazole-resistant phenotype (Table 1).

Multiple genes encoding efflux pumps associated with resistance to various antibiotics and antiseptics were detected, showing a heterogeneous distribution among the isolates (Table S1). Efflux pump genes linked to disinfectant resistance were also identified; the *amvA* and *adeC* genes were present in all isolates, whereas *qacEΔ1* (25/26, 96.2%) was present in all isolates but Acibau-26 (Figure 2 and Table S1).

Overall, genome-wide searches for antimicrobial-resistance genes provided a solid genetic basis to the XDR profile of all isolates, which shared a broad repertoire of AMR genes, including those implicated in disinfectant resistance.

### 2.2. cgMLST and Core Genome Phylogeny-Based Population Structure of A. baumannii Isolates

**Figure 3:**
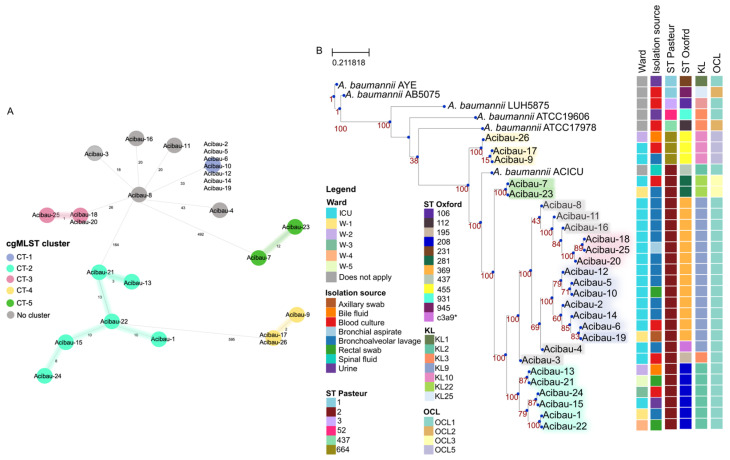
Phylogenetic and population structure of A. baumannii clinical isolates. (**A**) Minimum spanning tree of A. baumannii clinical isolates, constructed using a cluster distance threshold of 15 alleles. Nodes are colored according to CTs, and numbers on segments are the number of allelic differences. (**B**) Core genome-based maximum-likelihood phylogenetic tree including clinical isolates and reference A. baumannii strains. Bootstrap values are shown at branch nodes. Colored bars represent the isolate metadata, including isolation source, STs (Pasteur and Oxford schemes), capsular polysaccharide loci (KL), and lipooligosaccharide outer core loci (OCL). Strain shading is based on the cgMLST clusters defined in (**A**).

Core genome multi-locus sequence typing (cgMLST) was initially used to define the genetic relationships between isolates, which could be grouped into five distinct cluster types (CTs) (Figure 3A). CT-1 was composed of seven nearly identical isolates, whereas CT-2, CT-3, CT-4, and CT-5 comprised six, three, three, and two closely related isolates, respectively. Five isolates (Acibau-3, Acibau-4, Acibau-8, Acibau-11, and Acibau-16) could not be assigned to any CT according to cgMLST clustering parameters (Figure 3A).

Six out of seven CT-1 isolates (Acibau-2, Acibau-5, Acibau-6, Acibau-10, Acibau-12, and Acibau-14), all three CT-3 isolates (Acibau-18, Acibau-20, and Acibau-25) and two CT-4 isolates (Acibau-9 and Acibau-17) were from ICU patients, whereas Acibau-19 (CT-1), all CT-2 isolates and Acibau-26 originated from patients in different wards (Figure 3B).

The Pasteur MLST scheme assigned all isolates to the sequence type (ST) 2, with the exception of Acibau-9, Acibau-17, and Acibau-26, which belonged to ST664, a single-locus variant of ST2 (Figure 3B). Therefore, all isolates can be referred to as belonging to ICL II.

A clearly resolved pattern emerged when the Oxford MLST scheme was applied, showing that isolates clustering together according to the cgMLST scheme also shared identical or closely related Oxford STs. Accordingly, all STs defined for the 26 isolates according to the Oxford MLST scheme belong to or are closely related to clonal complex (CC) 208, which corresponds to ICL II (Figure 3B).

WGS-based phylogeny provided clear evidence of clustering of the 26 isolates together with the ICL II reference strain ACICU, and a clear separation from prototypic strains AYE and AB5075 (ICL I), LUH5875 (ICL III), ATCC19606 (the A. baumannii type strain), and ATCC17978. More importantly, an excellent match was noticed between groups defined by cgMLST and the phylogenomic tree topology. Indeed, CTs 1 to 5 were clearly separated in the phylogenomic tree, whereas the five isolates (Acibau-3, Acibau-4, Acibau-8, Acibau-11, and Acibau-16), which did not belong to any CT, were scattered in the phylogram (Figure 3B).

To associate phylotypes with outer surface antigenic structures, the capsular polysaccharide (KL) and outer-core lipooligosaccharide (OCL) loci were determined on a genomic basis. KL and OCL types were largely conserved within clades, with specific loci associated with individual Oxford STs and phylotypes (Figure 3B). In fact, all thirteen ST369 isolates (CTs 1 and 3, plus isolates Acibau-8, -11 and -16 not grouped in any CT) were KL9/OCL1, all six ST208 isolates (CT-2) were KL2/OCL1, all three ST455 isolates (CT-4) were KL10/OCL5, and both ST281 isolates (CT-5) were KL22/OCL3. Acibau-3 (ST195) and Acibau-4 (STc3a9), not grouped in any CT (Figure 3A), were KL3/OCL1 and KL9/OCL1, respectively (Figure 3B).

It can be argued that, although all 26 isolates belong to ICL II, they are distinctly subdivided into five interconnected clusters based on MLST and phylogenomics results. Inter-cluster diversity and intra-cluster similarity are well supported by the combination of genetic loci encoding variable surface structures, like the capsular polysaccharide and the lipooligosaccharide outer core.

### 2.3. Patristic Analysis of A. baumannii Isolates Based on Phylogenomic Data

The patristic analysis was conducted on a total of 339 WGSs of ICL II *A. baumannii* strains from Europe (*n* = 317) and North Africa (*n* = 22), retrieved from the Pathogenwatch platform. Core genome phylogeny was based on 31,249 single-nucleotide polymorphisms (SNPs), and the resulting tree topology highlighted well-supported phylogenetic clusters comprising isolates from INMI “Lazzaro Spallanzani” IRCCS and *A. baumannii* strains from Italy and/or European countries.

**Figure 4:**
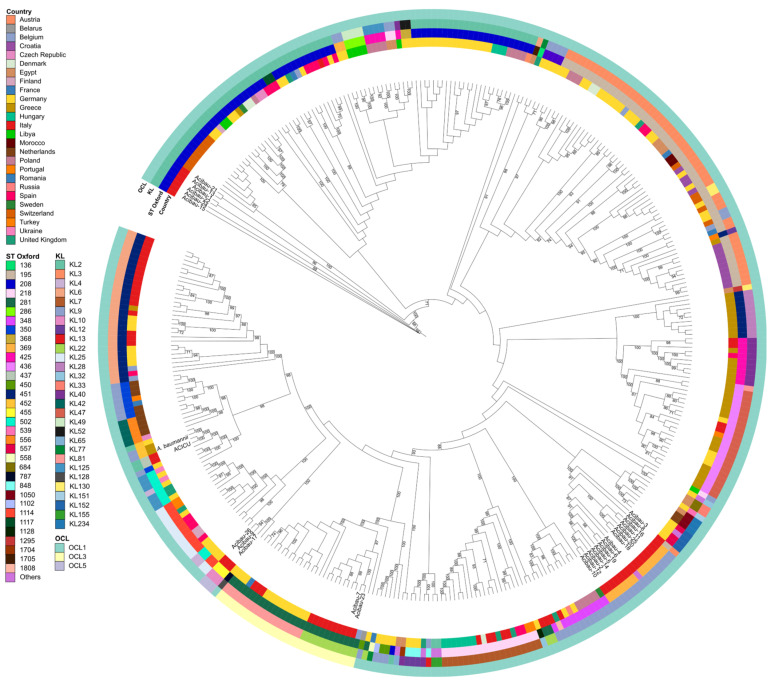
Maximum-likelihood cladogram inferred from core-genome comparison of 365 ICL II *A. baumannii* isolates from Europe (*n* = 317) and North Africa (*n* = 22), including the reference strain ACICU. Metadata tracks adjacent to the tree indicate country of isolation, ST Oxford, KL, and OCL. Bootstrap values above >70 are reported.

All CT-1 and CT-3 isolates were closely related to the Italian isolate SAMEA5229256 from Spedali Civili’s Hospital in Brescia (IT) (Figure 4, Table S2). All CT-5 isolates clustered with an Italian lineage, including SAMN08581087, SAMN08581088, SAMN08581095, SAMN08581096, SAMN08581099 and SAMN08581100 from Catania University Hospital (IT) and SAMN08398959 and SAMN08398936 from Agostino Gemelli University Hospital in Rome (IT), as well as with SAMN19493879 from Vrije University of Brussels (BE), all with robust bootstrap support. All CT-4 isolates were closely related to the German isolate SAMEA5396099, forming a separate subclade with high bootstrap confidence (Figure 4, Table S2). Lastly, all CT-2 isolates clustered in a separate group, unrelated to any strain from either Europe or North Africa.

### 2.4. Virulome Prediction

**Figure 5:**
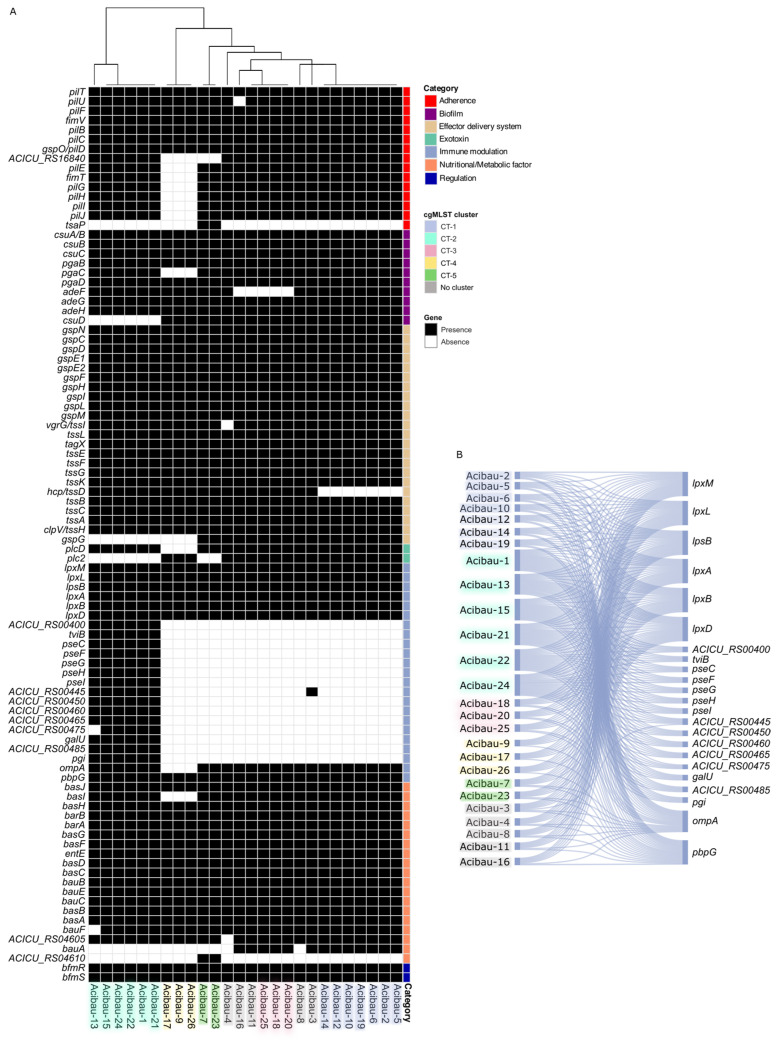
Virulence genes in 26 *A. baumannii* isolates from INMI “Lazzaro Spallanzani” IRCCS. (**A**) Distribution of virulence-related genes (black and white squares indicate gene presence and absence, respectively), grouped into functional categories, across isolates belonging to different CTs, as indicated in the legend. Functional categories and CTs are represented by distinct colours. (**B**) Sankey diagram highlighting the distribution of genes involved in immune modulation mechanisms among the isolates belonging to different CTs. Nodes and flows are colour-coded in blue to maintain consistency with the “Immune Modulation” functional category shown in Panel A. Panel B was generated using SankeyMATIC (https://sankeymatic.com/ (accessed on 13 March 2026).

Searches for the presence of virulence genes in the WGSs of the 26 isolates from INMI “Lazzaro Spallanzani” IRCCS were performed by direct comparison with 136 virulence genes categorized for the reference ICL II *A. baumannii* strain ACICU in the Virulence Factors Database (VFDB). Overall, 94 genes were detected in at least one isolate of the collection, highlighting the presence of a widespread core set of genes encoding for adherence factors, biofilm formation, exotoxins, nutritional and metabolic factors, effectors of delivery systems, and genes for immune modulation and virulence regulation (Figure 5A).

Hierarchical clustering of isolates based on virulome profile mirrored the subdivision obtained from phylogenomic analyses. CT-2 isolates were characterized by the broadest virulence gene repertoire, including a variety of immune modulation genes. In particular, CT-2 was the only cluster carrying the *pse* genes for the biogenesis of pseudaminic acid, a key component of KL type 2 *A. baumannii* capsule. However, compared with ACICU, CT-2 isolates lacked the biofilm-associated gene *csuD,* the effector delivery system gene *gspG*, and the phospholipase C *plc2* gene. Among CT-2 isolates, Acibau-13 also lacked the putative immune modulation gene *ACICU_RS00475* and the *bauF* gene for acinetobactin siderophore biosynthesis (Figure 5A). By comparison with the CT-2 virulome profile, all the other isolates lacked several immune modulation genes, except the *lpx* cluster for lipid A biosynthesis (*lpxA*, *lpxB*, *lpxD*, *lpxL*, and *lpxM*) and the *lpsB* gene.

Distinctive assemblages of virulence-related genes could also be observed for other CTs (Figure 5A,B). Isolates assigned to CT-4 carried a restricted repertoire of adherence-associated genes, which were limited to *pilT*, *pilU*, *pilF*, *fimV*, *pilB*, *pilC*, and *gspO/pilD*, and lacked the biofilm-associated *pgaC* gene, which encodes a glycosyltransferase responsible for the synthesis of poly-β-1,6-N-acetylglucosamine, a key exopolysaccharide component. In addition, they lack the *gspG* and *plcD* genes within the exotoxin category, *ompA* among immune modulation factors, and the acinetobactin biosynthetic gene *basI*. The CT-5 isolates lacked *ACICU_RS16840* among adherence-associated genes and *plc2* within the exotoxins category. Notably, they were the only isolates harbouring *ACICU_RS04610* among nutritional/metabolic factors, and the *tsaP* adherence factor. All CT-3 isolates lacked *adeF* within the biofilm category. Lastly, all CT-1 lacked *hcp*/*tssD* within the effector delivery system category.

The isolates not assigned to any CT disclosed a mosaic of different virulence-related genes. Acibau-4 was the only isolate lacking the *vgrG/tssI* gene for the type VI secretion system and the *ACICU_RS04605* gene from the nutritional/metabolic factor category. The virulence profile of Acibau-16 and Acibau-11 resembles that of CT-3 isolates, with the difference that Acibau-16 lacks the exotoxin gene *pilU*. The virulome of Acibau-8 and Acibau-3 resembled that of CT-1 isolates, though Acibau-8 lacked the acinetobactin receptor gene *bauA*, and Acibau-3 was the only isolate within the group carrying the immune modulation gene *ACICU_RS00445* (Figure 5A,B).

Immune modulation genes showed the most pronounced inter-strain variations, with *lpx* genes being present in the whole collection, whereas the KL 2 capsule genes (the *pse* cluster, *galU, pgi*) and a few other genes (the *tviB* siderophore receptor) appear unique to CT-2 (Figure 5A,B). Notably, all but CT-4 isolates carried the *ompA* gene, a key virulence factor [30].

## 3. Discussion

Continuous genomic surveillance of *A. baumannii* is increasingly recognized as a cornerstone for infection prevention and control in healthcare settings, particularly in the era of genomic epidemiology. The integration of WGS into routine surveillance has substantially improved the resolution of molecular typing and the ability to track high-risk lineages over time, and from the local to the global scale [6,13,16]. Moreover, WGS provides a complete, high-resolution view of the genetic makeup of AMR and virulence, which can be correlated with bacterial pathogenicity.

Within the hospital infection surveillance system of INMI “Lazzaro Spallanzani” IRCCS, the molecular epidemiology and genetic basis of AMR were investigated in a collection of 26 CRAB isolates collected over one year from patients treated in different hospital wards. All isolates exhibited the same AMR pattern and could be classified as XDR, being susceptible only to colistin and cefiderocol. Despite their identity at the resistance-phenotype level, some differences were observed in the repertoire of AMR genes carried by the isolates, as inferred from interrogation of multiple databases. It has been argued that the use of a multi-database strategy for the annotation of resistance genes enhances confidence and comprehensiveness in defining the genetic basis of resistance for both epidemiological and clinical applications, given the different design and intended purposes of individual databases [31]. Indeed, CARD incorporates a broad range of resistance determinants, regardless of their clinical relevance, making it suitable for exploratory genomic analyses of complex resistomes; ResFinder adopts a more conservative approach for gene detection, focusing on resistance genes of direct clinical relevance; AMRFinder complements the information from previous databases by providing a hierarchical ontology structure to classify AMR determinants. By combining the results from the three databases, the homogeneous pattern of phenotypic resistance to β-lactams, carbapenems, aminoglycosides, fosfomycin, fluoroquinolones, and trimethoprim-sulfamethoxazole was fully supported by the detection of the corresponding resistance genes in all isolates. Despite some inter-isolate differences in the resistome, a good correlation between phylogenomic architecture and resistome structure was observed (Figure S1). Indeed, hierarchical clustering of isolates based on presence/absence of resistance genes showed that all CT-1 isolates lacked *sul2*, CT-3 isolates lacked both *aph(3′)-Ia* and *catB8*, while CT-5 isolates carried the rare *bla*_OXA-82_ allele (Figure S1). More importantly, the resistome of all isolates was invariably composed of a conserved backbone of AMR determinants, including *ant(3^II^)-IIa*, *bla*_OXA-23_, *bla*_OXA-51-like_, *gyrA*S81L, *parC*S64L, *abaF*, *sul* (*1* or *2*), and a variety of efflux pump genes contributing to both antimicrobial and disinfectant resistance. Altogether, these determinants fully explain the conserved resistance profile of the entire collection, as inferred from MIC data (Table 1). The dominance of *bla*_OXA-23_ supports the well-established evidence that this carbapenemase represents the primary driver of carbapenem resistance in *A. baumannii* worldwide, particularly among isolates belonging to the ICL II [17] (Table 1 and Table S1). Among the *bla*_OXA-51-like_ alleles, the *bla*_OXA-82_ variant was exclusively detected in CT-5 isolates by both CARD and AMRFinder, while ResFinder gave the erroneous assignment to *bla*_OXA-66,_ as verified by a punctual check of coding sequences. This exemplifies the importance of a multi-database approach for the correct and exhaustive identification of resistance genes. It should be noticed that *bla*_OXA-82_ differs from *bla*_OXA-66_ for only 2 out of 825 nucleotides (99.8% identity), and it can arise from *bla*_OXA-66_ by intra-clonal evolution [32]. Although *bla*_OXA-51-like_ genes can confer carbapenem resistance when preceded by IS*Aba1* [33], no IS*Aba1* element was detected upstream of the *bla*_OXA-51-like_ genes in any of the isolates.

All isolates were susceptible to colistin and cefiderocol, but these findings must be interpreted cautiously (Table 1), since *A. baumannii* can rapidly develop cefiderocol-resistance during therapy, often through mutations or insertion sequences affecting TonB-dependent siderophore receptor genes such as *pirA* and *puiA* or some stress-response genes [34,35,36]. Similarly, colistin resistance is still relatively uncommon, but it can emerge through modifications or loss of lipopolysaccharide mediated by mutations in the *lpx* or *pmr* pathways, with important implications for bacterial fitness and therapeutic outcomes [37,38,39]. Even in settings where susceptibility is maintained, the risk of emerging resistance underscores the importance of caution and continued monitoring when using last-line agents.

The WGS analysis of the 26 CRAB isolates revealed a structured yet heterogeneous population, consistent with the well-documented genomic plasticity and intra-clonal diversity ICL II *A. baumannii* circulating in hospitals of the Rome area and abroad [40,41,42,43,44,45]. This result is consistent with genomic analyses demonstrating that ST2 is composed of several distinct subclones, endowed with different accessory gene profiles, coexisting within the same healthcare setting [46].

The ICL II lineage is the main driver of CRAB dissemination and has been repeatedly associated with extensive AMR, environmental persistence, and the ability to cause prolonged endemic circulation and outbreaks in critical care settings [11]. While all isolates could be assigned to ICL II, cgMLST enabled high-resolution discrimination between groups of isolates with the identification of five CTs, plus five isolates that could not be assigned to any CT (Figure 3A; Figure S2). Coherently, the Oxford MLST scheme and whole genome-based phylogeny differentiated the 26 *A. baumannii* isolates into five groups closely matching those determined by the cgMLST, whereas the Pasteur MLST scheme was less discriminatory. An interesting point is that phylogenetic and phylogenomic clustering confirmed that a close genomic similarity was not consistently associated with the source of the organism, since nearly identical isolates originated from different wards during time (Figure 3B, Figure S2), depicting a scenario compatible with both local circulation of endemic clones and, plausibly, the introduction of CRAB strains over the study period. While all six different phylotypes were detected in the ICU, only CT-1 and all unassigned isolates were detected during the whole surveillance period. The CT-2 isolates were isolated from all wards in the hospital in the different years, whereas CT-3 isolates originated only from ICU patients and were introduced in 2024 together with CT-4. All CT-1, CT-4, and CT-5 were detected in a single ward, besides being also present in the ICU (Figure S2). This mosaic of multiple ICL II clonal types from a single healthcare facility is consistent with reports describing inter-hospital transmission as a key mechanism sustaining regional circulation of CRABs, particularly for ICL II-associated lineages [6]. Most patients were colonized upon admission, consistent with former evidence that CRAB is frequently imported into hospitals via patient transfer [47,48,49,50]. Of note, colonization has been reported to precede subsequent infection and be associated with adverse outcomes in critically ill patients, highlighting the importance of early detection and targeted prevention strategies [51].

Several variants of the capsular locus were identified among the CRAB genomes, including KL9, KL2, KL3, KL10, and KL22, with KL9 being the most frequent. KL9 and KL10 gene clusters encode capsules composed mainly of neutral hexoses and N-acetylated sugars, whereas KL3 and KL22 include genes for the synthesis of acidic sugars such as glucuronic acid, which confer a negative charge to the capsule [52]. KL2 is among the most widespread capsule types in XDR clinical isolates, and encodes the enzymes implicated in pseudaminic or legionaminic acid biosynthesis [53,54]. In our dataset, the KL2 locus was identified exclusively in CT-2 isolates; consistently, only CT-2 isolates carried the hypervariable region for pseudaminic acid biosynthesis (*pse*). The combined analysis of KL and OCL for capsular and outer-core composition revealed a strong concordance with the phylogenetic structure of the isolates. The conserved KL10/OCL5 and KL22/OCL3 combinations in ST455 and ST281 isolates, respectively, reflect the tendency of these antigen-encoding loci to be stably maintained within individual clones.

Virulome profiling of the whole isolate collection unveiled a conserved core of genes implicated in envelope structure, secretion systems, and environmental persistence, consistent with the multi-factorial nature of *A. baumannii* pathogenicity [2,55]. At the same time, cluster-specific differences were observed, including the absence in certain CTs of subsets of genes implicated in adherence, biofilm formation, or host interaction. Among genes of immune modulation group, only those of the *lpx* operon were invariably present in all isolates, while a variety of immunomodulatory factors were not detected in CT-1 isolates, and all CT-4 isolates lacked the *ompA* porin gene, encoding the OmpA multifunctional outer membrane protein which acts as a key virulence factor involved in adhesion, biofilm formation, immune interaction, and host cell damage [30]. These findings support the idea that *A. baumannii* preserves its infectivity and pathogenic potential, despite displaying some lineage-specific genetic diversity in virulence traits [56,57].

The patristic analysis suggests that the 26 CRAB isolates from INMI “Lazzaro Spallanzani” IRCCS are not the result of local evolutionary events but rather belong to established lineages circulating across Europe. The clustering of multiple CTs with strains from different countries, particularly from Italy and central Europe (Germany and Belgium), supports the notion that ICL II *A. baumannii* is composed of several geographically widespread clones. This pattern is consistent with the well-documented ability of ICL II *A. baumannii* to maintain genomic stability while disseminating across healthcare settings [58,59].

Overall, our findings depict a heterogeneous genomic landscape dominated by ICL II-related CRABs, characterized by a conserved backbone of resistance genes shared by different phylogenetic groups endowed with specific genetic signatures. These results underscore the importance of robust genomic surveillance frameworks capable of detecting relevant genomic changes and supporting evidence-based infection prevention and antimicrobial stewardship strategies.

## 4. Limitations and Perspectives

The relatively small number of isolates and the restricted sampling period limit inferences about the long-term evolutionary dynamics of this study, while the monocentric design hampers the generalization of the findings to broader national contexts. Furthermore, the absence of phenotypic analyses involving, e.g., adhesion and biofilm formation, cytotoxicity, virulence in animal models, and environmental persistence, constrains the interpretation of how genomic traits translate into the potential pathogenicity of CRAB isolates. Future studies integrating genomics with phenotypic and clinical data will be essential to refine predictive models and fully exploit the potential of WGS-based surveillance. The integration of multicenter sampling, temporal monitoring, and genotype–phenotype correlations will help refine epidemiological models and better predict the emergence and dissemination of novel CRAB variants.

## 5. Materials and Methods

### 5.1. Collection and Phenotypic Characterization of the Isolates

Samples were collected over one year, between September 2023 and September 2024, from 26 patients admitted to the wards of the INMI “Lazzaro Spallanzani” IRCCS. In addition to carbapenem resistance, the selection criterion for inclusion in the CRAB collection was the availability of complete microbiological and clinical metadata for each isolate. To ensure a non-redundant dataset, only the index isolate from each patient was considered. The selected CRAB isolates were from patients aged 38 to 80 years (median age: 64).

The samples were analyzed via culture method to isolate bacteria, using Chromatic^TM^ CRE Agar Base (Liofilchem srl, Roseto degli Abruzzi, Italy), incubated at 37 °C under aerobic conditions. The growing colonies were identified using the MALDI-TOF Biotyper Sirius System (Bruker Daltonics, Bremen, Germany) and MBT Compass software (v4.2). Antimicrobial susceptibility testing (AST) of the isolates was subsequently performed via Phoenix panel (NMIC-474) run on the Phoenix system PMICR 96 (Becton Dickinson Diagnostics, San Jose, CA, USA). The results of the MICs were interpreted according to the recent guidelines established by EUCAST 2024 (v. 14.0) [23].

### 5.2. DNA Isolation, Whole-Genome Sequencing (WGS), cgMLST, and Phylogenomic Analyses

Bacterial DNA was extracted using the Qiamp DNA mini-Kit (Qiagen, Hilden, Germany) and subsequently quantified with the Invitrogen^TM^ 4 Fluorometer (Thermo Fisher Scientific Inc., Monza, Italy).

Libraries were prepared using the Nextera DNA Library Prep Kit, and sequencing was performed using the MiSeq sequencer (Illumina Inc., San Diego, CA, USA). The sequences obtained were preliminarily processed by the Illumina BaseSpace system and converted to FASTQ files for subsequent bioinformatics analysis.

Raw sequencing reads were quality-checked and trimmed using Trimmomatic [60] to remove adapters and low-quality bases. The resulting high-quality reads were assembled de novo with SPAdes [61]. Gene prediction was performed with Prodigal [62].

Resistance genes were identified using ABRicate (v.1.4.0) by screening the AMRFinder [24] database. Furthermore, resistance genes were identified by interrogating the CARD [25] and ResFinder (v 4.7.2) [26] databases via the online platforms (available at https://card.mcmaster.ca/analyze/blast (accessed on 26 February 2026) and https://genepi.food.dtu.dk/resfinder (accessed on 24 February 2026), respectively). A minimum sequence identity threshold of 100.0 and an alignment length requirement of >98.0% were applied. The prediction of the pathogenicity of a bacterium toward the human host was obtained using the online tool PathogenFinder (v1.1) (available at https://cge.food.dtu.dk/services/PathogenFinder/, accessed on 29 April 2025). When needed, whole genome sequences were manually inspected to detect the presence of *A. baumannii* insertion sequences (*IS*) and verify ambiguous or discordant AMR gene assignments inferred from multiple database searches.

STs were identified using the MLST tool (available at https://github.com/tseemann/mlst, accessed on 5 February 2026), applying both the Pasteur and Oxford *A. baumannii* MLST schemes; allelic profiles and ST assignments were retrieved from the PubMLST *A. baumannii* database (https://pubmlst.org/abaumannii/, accessed on 5 February 2026), which served as the reference repository for both schemes.

To further investigate the genetic relationships among isolates and to identify the CTs, we used the WGS-based cgMLST scheme (v 1.0), using the Ridom SeqSphere+ software (Ridom GmbH, Münster, Germany) with default parameters. The cgMLST scheme included 2390 core genes and was built using the reference genome ACICU (accession number NC_010611.1), following the standardized procedure reported in the software manual [63]. Allelic profiles were determined for each isolate and used to calculate genetic distances between genomes. These distances were then used to generate a minimum spanning tree (MST) to visualize the genetic relationships among isolates. Clonal relationships were defined based on allelic differences, applying a threshold of ≤15 allelic differences to identify clusters of closely related isolates [64].

Population structure and phylogenetic relationships among the isolates and six reference strains were inferred using WGS data. The reference strains used in the analysis were *A. baumannii* AB5075 (NZ_JHUI01000007), *A. baumannii* ACICU (NC_010611), *A. baumannii* ATCC17978 (NZ_CACVBA010000001), *A. baumannii* ATCC19606 (NZ_CP058289), *A. baumannii* AYE (NC_010410), and *A. baumannii* LUH5875 (DADAQT010000001).

Core genome alignments had a total of 108,921 high-quality SNPs identified by Snippy (v4.6.0) using *A. baumannii* AYE (GenBank accession no. NC_010410) as reference.

A maximum-likelihood phylogenetic tree was constructed from the core genome alignment using RAxML (v8.2.4) [65], and branch support was assessed by bootstrap analysis. The resulting phylogeny was visualized and annotated using the ETE3 Python module (v.3.1.3) [66], which allowed for an integrated representation of evolutionary relationships and associated metadata.

Population structure and phylogenetic relationships among *A. baumannii* ST2 isolates from Europe and North Africa were inferred using WGS data. A total of 339 genomes, including 317 European and 22 North African isolates, were retrieved from the Pathogenwatch platform together with their associated metadata. Core genome SNPs were identified using Snippy (v4.6.0), with *A. baumannii* ACICU (GenBank accession no. CP000863) used as the reference genome. The resulting core genome alignment included 31,249 high-quality SNPs. A maximum-likelihood phylogenetic tree was reconstructed from the core genome alignment using RAxML (v8.2.4), and branch support was evaluated by bootstrap analysis [65]. The resulting phylogeny was visualized, annotated, and integrated with epidemiological and geographic metadata using the Interactive Tree of Life (iTOL) web server (available at https://itol.embl.de/, accessed on 25 February 2026) [67], enabling the graphical representation of evolutionary relationships and strain distribution across regions.

### 5.3. Annotation of Virulence Factor Genes

The annotation of the virulence was performed using the Virulence Factors Database (VFDB, available at https://www.mgc.ac.cn/VFs/, accessed on 30 January 2026), after restricting the reference dataset to sequences belonging to A. baumannii ACICU. The curation of the virulence genes was based on their established roles in adhesion, biofilm formation, and host interaction, which are recognized as key determinants of A. baumannii pathogenicity [30,68]. The selection was applied directly at the database level, before sequence comparison. Nucleotide sequences from the filtered VFDB dataset were used to build a custom BLAST database using makeblastdb (v.2.17.0). Genome-derived nucleotide sequences were then aligned against this database using blastn (v.2.17.0). To ensure highly stringent and unambiguous annotations, BLASTn results were filtered to retain only hits showing 100.0% sequence identity and 100.0% alignment coverage across the query sequence. Valid hits were subsequently grouped according to their functional virulence categories as defined in VFDB. The hierarchical clustering of the isolates was inferred using Euclidean distance [69].

### 5.4. Data Availability

All raw reads generated were submitted to the Sequence Read Archive (SRA) under the BioProject ID PRJNA1414926.

## 6. Conclusions

In conclusion, this study provides a high-resolution snapshot of the current CRABs genomic landscape, illustrating the structured diversity and evolutionary dynamism that characterize high-risk lineages in a well-defined clinical setting. Interrogation of multiple databases was essential for accurate annotation of resistance genes and could exhaustively explain the XDR phenotype of CRABs. While all isolates belonged to the high-risk ICL II, high-resolution phylogenomic investigations resolved them into five clonal types and a few singletons, confirming that within ICL II, there is considerable genomic diversity. Nearly identical isolates were recovered from different wards over time, suggesting local endemic circulation alongside repeated introductions from external healthcare facilities. Patristic analysis linked the study clusters to ICL II strains from other Italian cities and European countries, indicating that these CRAB lineages are part of broader, cross-border dissemination. A core set of virulence-related genes was conserved across all isolates, but cluster-specific differences were identified. Beyond the characterization of evolutionary, resistance, and virulence traits in our isolate collection, our findings underscore the importance of continuous genomic surveillance as an essential tool for tracking high-risk *A. baumannii* clones, understanding their transmission dynamics, and supporting infection control strategies and antimicrobial stewardship.

## Figures and Tables

**Table 1:** Antimicrobial susceptibility profile of CRAB isolates.

				Antibiotic MIC (mg/L) and Interpretation									
Isolate Designation	Isolation Date (m.d.y)	Source	IMP	MEM	ETP	GEN	AMK	CIP	SXT	COL	FDC	FOS	TGC *
**Acibau-1**	09.28.2023	BAL	>8, R	>8, R	>1, R	>4, R	>8, R	>1, R	>4/76, R	0.5, S	2, S	128, R	2
**Acibau-2**	11.02.2023	BAL	>8, R	>8, R	>1, R	>4, R	>8, R	>1, R	>4/76, R	0.25, S	0.5, S	256, R	2
**Acibau-3**	11.10.2023	Blood Culture	>8, R	>8, R	>1, R	>4, R	>8, R	>1, R	>4/76, R	1, S	0.25, S	256, R	4
**Acibau-4**	11.27.2023	BAL	>8, R	>8, R	>1, R	>4, R	>8, R	>1, R	>4/76, R	0.25, S	0.5, S	128, R	2
**Acibau-5**	11.27.2023	BAL	>8, R	>8, R	>1, R	>4, R	>8, R	>1, R	>4/76, R	0.25, S	0.5, S	128, R	2
**Acibau-6**	12.02.2023	Blood Culture	>8, R	>8, R	>1, R	>4, R	>8, R	>1, R	>4/76, R	0.25, S	0.5, S	128, R	1
**Acibau-7**	12.06.2023	Blood Culture	>8, R	>8, R	>1, R	>4, R	>8, R	>1, R	>4/76, R	0.5, S	1, S	128, R	2
**Acibau-8**	08.14.2023	BAL	>8, R	>8, R	>1, R	>4, R	>8, R	>1, R	>4/76, R	1, S	1, S	>256, R	2
**Acibau-9**	01.17.2024	BAL	>8, R	>8, R	>1, R	>4, R	>8, R	>1, R	>4/76, R	0.5, S	2, S	128, R	2
**Acibau-10**	01.22.2024	Rectal swab	>8, R	>8, R	>1, R	>4, R	>8, R	>1, R	>4/76, R	0.5, S	0.25, S	128, R	2
**Acibau-11**	01.29.2024	BAL	>8, R	>8, R	>1, R	>4, R	>8, R	>1, R	>4/76, R	0.5, S	0.25, S	128, R	2
**Acibau-12**	01.29.2024	BAL	>8, R	>8, R	>1, R	>4, R	>8, R	>1, R	>4/76, R	0.5, S	0.25, S	128, R	2
**Acibau-13**	02.26.2024	Bile fluid	>8, R	>8, R	>1, R	>4, R	>8, R	>1, R	>4/76, R	0.5, S	2, S	128, R	2
**Acibau-14**	03.14.2024	BAL	>8, R	>8, R	>1, R	>4, R	>8, R	>1, R	>4/76, R	1, S	0.25, S	128, R	2
**Acibau-15**	04.29.2024	Urine	>8, R	>8, R	>1, R	>4, R	>8, R	>1, R	>4/76, R	0.5, S	1, S	>256, R	2
**Acibau-16**	05.13.2024	BAL	>8, R	>8, R	>1, R	>4, R	>8, R	>1, R	>4/76, R	0.5, S	0.25, S	>256, R	1
**Acibau-17**	07.15.2024	Blood Culture	>8, R	>8, R	>1, R	>4, R	>8, R	>1, R	>4/76, R	2, S	2, S	>256, R	2
**Acibau-18**	08.11.2024	BAL	>8, R	>8, R	>1, R	>4, R	>8, R	>1, R	>4/76, R	1, S	1, S	>256, R	2
**Acibau-19**	08.12.2024	Axillary swab	>8, R	>8, R	>1, R	>4, R	>8, R	>1, R	>4/76, R	0.5, S	2, S	>256, R	2
**Acibau-20**	08.16.2024	BAL	>8, R	>8, R	>1, R	>4, R	>8, R	>1, R	>4/76, R	0.5, S	2, S	256, R	2
**Acibau-21**	08.17.2024	Rectal swab	>8, R	>8, R	>1, R	>4, R	>8, R	>1, R	>4/76, R	0.5, S	0.25, S	128, R	2
**Acibau-22**	08.17.2024	Rectal swab	>8, R	>8, R	>1, R	>4, R	>8, R	>1, R	>4/76, R	1, S	0.25, S	128, R	2
**Acibau-23**	08.20.2024	BAL	≤4, S	>8, R	>1, R	>4, R	>8, R	>1, R	>4/76, R	2, S	2, S	128, R	2
**Acibau-24**	08.28.2024	Blood Culture	>8, R	>8, R	>1, R	>4, R	>8, R	>1, R	>4/76, R	0.5, S	2, S	128, R	2
**Acibau-25**	09.02.2024	BAS	>8, R	>8, R	>1, R	>4, R	>8, R	>1, R	>4/76, R	1, S	2, S	128, R	2
**Acibau-26**	09.09.2024	Bile fluid	>8, R	>8, R	>1, R	>4, R	>8, R	>1, R	>4/76, R	0.25, S	0.25, S	128, R	2

Abbreviations: IPM: imipenem; MEM: meropenem; ETP: ertapenem; GEN: gentamycin; AMK: amikacin; CIP: ciprofloxacin; SXT: trimethoprim-sulfamethoxazole; COL: colistin; FDC: cefiderocol; FOS: fosfomycin; TGC: tigecycline. R: resistant; S: susceptible. Phenotypic interpretation was performed according to EUCAST clinical breakpoint tables 2024 (v. 14.0) [23]. * Note: for tigecycline, only MIC values are reported as no clinical breakpoints for *Acinetobacter* spp. have been established by EUCAST. Interpretative criteria are currently unavailable for this species–drug combination.

## Data Availability

The data can be found in the Excel database, created ad hoc and archived at the authors’ institution (INMI “L. Spallanzani” IRCCS, Rome, Italy).

## Supplementary Materials

The following supporting information can be downloaded at: https://www.mdpi.com/article/10.3390/antibiotics15050464/s1, Table S1: Overview of antimicrobial resistance genes detected in the 26 *Acinetobacter baumannii* isolates using AMRFinder, CARD, and ResFinder. Table S2: Metadata retrieved from Pathogenwatch for the 339 *Acinetobacter baumannii* isolates included in the patristic analysis. Figure S1: Hierarchical clustering of 26 CRAB isolates from INMI “Lazzaro Spallanzani” IRCCS according to their repertoire of AMR genes, inferred from WGS analysis. Figure S2: Distribution of *A. baumannii* isolates across wards and cluster types (CTs).

## Abbreviations

The following abbreviations are used in this manuscript:

AMK	Amikacin
AMR	Antimicrobial resistance
AST	Antimicrobial susceptibility testing
CC	Clonal complex
cgMLST	core genome Multi-Locus Sequence Typing
CIP	Ciprofloxacin
COL	Colistin
CRAB	Carbapenem-Resistant A. baumannii
CT	Cluster Type
EPT	Ertapenem
FDC	Cefiderocol
FOS	Fosfomycin
GEN	Gentamycin
ICL II	International Clone II
ICUs	Intensive Care Units
INMI	National Institute for Infectious Diseases
IPM	Imipenem
IRCCS	Istituto di Ricovero e Cura a Carattere Scientifico
IS	Insertion sequences
KL	Capsular polysaccharide locus
MEM	Meropenem
MICs	Minimum Inhibitory Concentrations
MST	Minimum Spanning Tree
OCL	Lipooligosaccharide outer core locus
R	Resistant
S	Susceptible
SNPs	Single-Nucleotide Polymorphisms
SRA	Sequence Read Archive
ST	Sequence Type
SXT	Trimethoprim-sulfamethoxazole
TGC	Tygeciclin
VFDB	Virulence Factors Database
W-1	Post-surgical ICU
W-2	Hepatology Unit
W-3	Respiratory Diseases Unit
W-4	Immune Systemic Infectious Diseases Unit
W-5	High-Intensity Care Infectious Unit
WGS	Whole-Genome Sequencing
WHO	World Health Organization
XDR	Extensively drug-resistant
