# Supplementary material for: High-Resolution Genomic Surveillance of Carbapenem-Resistant Acinetobacter baumannii: IC-2 Clonal Diversity, Resistance Determinants, and Virulence Signatures
Source: Antibiotics (Basel). 2026 May 4;15(5):464. doi: 10.3390/antibiotics15050464 (PMC13203267; doi:10.3390/antibiotics15050464)
Supplement: Supplementary file 1 [file antibiotics-15-00464-s001.zip › Supplementary Figures S1 and S2 Antibiotics-4276808.pdf]

Supplementary material for

High-Resolution Genomic Surveillance of Carbapenem-Resistant *Acinetobacter baumannii*: IC-2 Clonal Diversity, Resistance Determinants, and Virulence Signatures

Arianna Basile [1,+], Valentina Antonelli [2,+], Claudia Rotondo [2,*], Michele Properzi [2], Francesco Messina [2], Silvia D'Arezzo [2], Valentina Dimartino [2], Ivano Petriccione [2], Laura Loiacono [3], Maria Grazia Bocci [4], Giulia Capecchi [4,*], Alessia Arcangeli [5], Alessandra Marani [6], Filippo Pasquale Riggio [1], Massimiliano Lucidi [1], Francesco Imperi [1], Paolo Visca [1,7,8,+], Carla Fontana [2,+]

[1] Department of Science, Roma Tre University, Rome, Italy; arianna.basile@uniroma3.it; filippopasquale.riggio@uniroma3.it; massimiliano.lucidi@uniroma3.it; francesco.imperi@uniroma3.it; paolo.visca@uniroma3.it
[2] Microbiology and Biobank Unit, National Institute for Infectious Diseases "Lazzaro Spallanzani", IRCCS, 00149 Rome, Italy; valentina.antonelli@inmi.it; claudia.rotondo@inmi.it; michele.properzi@inmi.it; mss.francesco1984@gmail.com; silvia.darezzo@inmi.it; valentina.dimartino@inmi.it; ivano.petriccione@inmi.it; carla.fontana@inmi.it
[3] Clinical and Research Infectious Disease Department, National Institute for Infectious Diseases "Lazzaro Spallanzani", IRCCS, 00149 Rome, Italy; laura.loiacono@inmi.it
[4] Intensive Care Unit, National Institute for Infectious Diseases "Lazzaro Spallanzani", IRCCS, 00149 Rome, Italy; mariagrazia.bocci@inmi.it; giulia.capecchi@inmi.it
[5] Clinical Risk Management, National Institute for Infectious Diseases "Lazzaro Spallanzani", IRCCS, 00149 Rome, Italy; alessandra.arcangeli@inmi.it
[6] Health Direction, National Institute for Infectious Diseases "Lazzaro Spallanzani", IRCCS, 00149 Rome, Italy; alessandra.marani@inmi.it
[7] NBFC, National Biodiversity Future Center, Palermo, Italy; paolo.visca@uniroma3.it
[8] Santa Lucia Foundation IRCCS, Rome, Italy; paolo.visca@uniroma3.it
* Correspondence: C.R. (claudia.rotondo@inmi.it); G.C. (giulia.capecchi@inmi.it)
[+] These authors equally contributed to this work

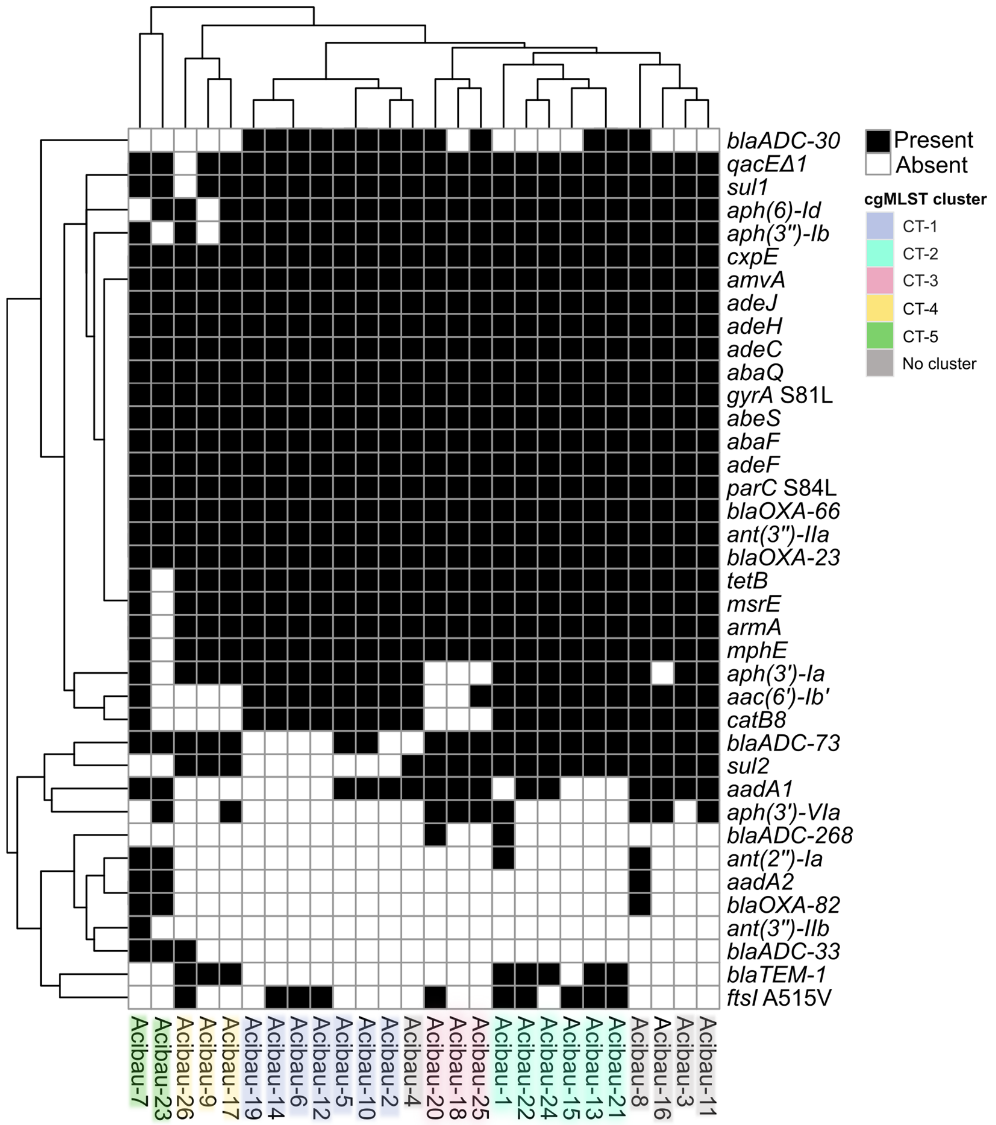

Figure S1: Hierarchical clustering of 26 CRAB isolates from INMI "Lazzaro Spallanzani" IRCCS according to their repertoire of AMR genes, inferred from WGS analysis. The heatmap shows the presence (black) or absence (white) of the individual antibiotic resistance genes listed on the right side of the figure. Results were binarized such that any evidence from one or more databases was considered as gene presence.

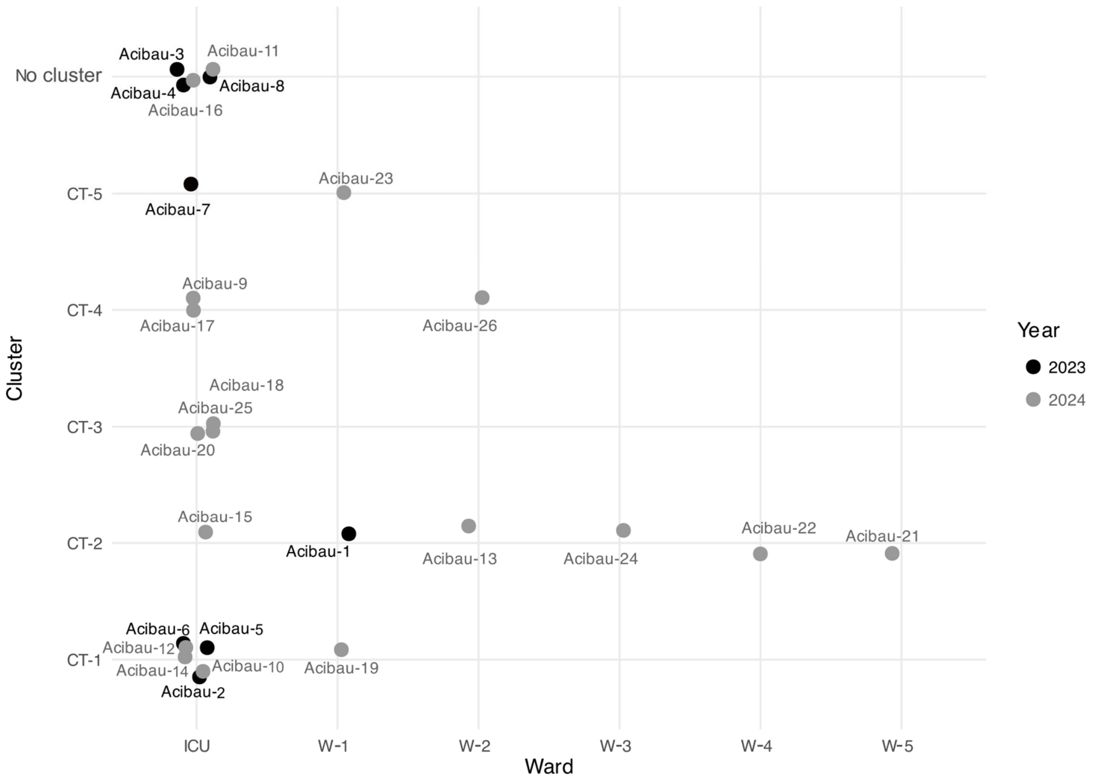

Figure S2: Distribution of *A. baumannii* isolates across wards and CTs. Each point is a single isolate located in the diagram according to the patient's ward and CT. Points are differentiated by year of isolation (2023, black; 2024, grey). A small jitter was applied to avoid overlap between isolates sharing the same ward–cluster combination.
